# Comparing the Degradation Potential of Copper(II), Iron(II), Iron(III) Oxides, and Their Composite Nanoparticles in a Heterogeneous Photo-Fenton System

**DOI:** 10.3390/nano11010225

**Published:** 2021-01-16

**Authors:** Asfandyar Khan, Zsolt Valicsek, Ottó Horváth

**Affiliations:** 1 Department of General and Inorganic Chemistry, Faculty of Engineering, University of Pannonia, 8200 Veszprém, Hungary; asfandyarkhan100@gmail.com (A.K.); valicsek@almos.uni-pannon.hu (Z.V.); 2 Department of Textile Processing, National Textile University, Faisalabad, Punjab 37610, Pakistan

**Keywords:** heterogeneous photo-Fenton system, copper oxide, iron oxides, copper ferrite, simple precipitation, photodegradation

## Abstract

Heterogeneous photo-Fenton systems offer efficient solutions for the treatment of wastewaters in the textile industry. This study investigated the fabrication and structural characterization of novel peculiar-shaped Cu^II^O, Fe^III^_2_O_3_, and Fe^II^O nanoparticles (NPs) compared to the properties of the iron(II)-doped copper ferrite Cu^II^_0.4_Fe^II^_0.6_Fe^III^_2_O_4_. The photocatalytic efficiencies of these NPs and the composite of the simple oxides (Cu^II^O/Fe^II^O/Fe^III^_2_O_3_) regarding the degradation of methylene blue (MB) and rhodamine B (RhB) as model dyes were also determined. The catalysts were synthesized via simple co-precipitation and calcination technique. X-ray diffractometry (XRD), scanning electron microscopy (SEM), and diffuse reflectance spectroscopy (DRS) were utilized for structural characterization. The structure of Cu^II^O was bead-like connected into threads, Fe^III^_2_O_3_ was rod-like, while Fe^II^O pallet-like, with average crystallite sizes of 18.9, 36.9, and 37.1 nm, respectively. The highest degradation efficiency was achieved by Cu^II^O for RhB and by Cu^II^_0.4_Fe^II^_0.6_Fe^III^_2_O_4_ for MB. The Cu^II^O/Fe^II^O/Fe^III^_2_O_3_ composite proved to be the second-best catalyst in both cases, with excellent reusability. Hence, these NPs can be successfully applied as heterogeneous photo-Fenton catalysts for the removal of hazardous pollutants. Moreover, the simple metal oxides and the iron(II)-doped copper ferrite displayed a sufficient antibacterial activity against Gram-negative *Vibrio fischeri*.

## 1. Introduction

The increase in the global world population results in urbanization as well as industrialization, which ultimately sources increase in water pollution. One of the major contributors to water pollution across the world is the textile industry because most of its factories drain their effluents containing hazardous and toxic by-products without further treatment into bio-compatible products [1,2]. Rhodamine B, methylene blue, Congo red, and methyl orange are some of the major hazardous and toxic pollutants. The degradation of such complex organic compounds into smaller bio-compatible molecules is necessary for a greener environment, but they hardly decay in natural ways [3]. Treatments of synthetic [4] and municipal wastewaters [5] have recently been explored. Photocatalytic degradation was introduced in the 1970s, being one of the deeply investigated methods in this respect [6,7]. Sunlight is a cheap, abundant, and renewable source of energy, and can be utilized in the photocatalytic processes [8].

Nanoparticles deliver interesting optical and electronic properties due to their dimensions, which make them ideal candidates in the development of functional nanostructures for their applications in catalysis, optoelectronics, drug delivery, and chemical sensors or biosensors, etc. Semiconductor nanoparticles, such as titanium dioxide (TiO_2_), zinc oxide (ZnO), cadmium sulfide (CdS), copper oxide (Cu^II^O), and iron oxide (Fe^III^_2_O_3_), have attracted attention for their successful utilization as photocatalysts in the field of environmental purification [3,9,10,11,12,13]. Photocatalysts, also known as photochemical catalysts, work similarly to chlorophylls in the process of photosynthesis. Photo-induced reactions take place at the catalyst surface as results of the generation of electron–hole pair, which diffuses out to the photocatalyst surface and takes part in redox reactions transforming the surrounding water or oxygen molecules into free radicals with super strong oxidization potential. Hence, photocatalysts may be used in killing germs, pollen, bacteria, viruses, and epiphytes and may mineralize hazardous compounds including dyes, pigments, aryl compounds, and amine compounds without producing secondary pollution [9,14,15].

Literature reported numerous simple oxide semiconductor materials as well as composites in photocatalytic processes, e.g., TiO_2_ [16], NiFe_2_O_4_ [11], ZnFe_2_O_4_ [10], and CuFe_2_O_4_ [17] in the field of environmental remediation. TiO_2_ has a disadvantage of an inherently wide band gap (3.2 eV), which makes it able to utilize only a small portion (4%) of the solar spectrum. Application of composites, such as TiO_2_-SiO_2_ [18], CNT/TiO_2_, CNT/TiO_2_-SiO_2_, Au/TiO_2_, CNT-Au/TiO_2_, and CNT-Au/TiO_2_-SiO_2_ (where CNT stands for carbon nanotubes), resulted in synergistic effects on the photocatalytic performance of the individual components [19]. Copper oxide (Cu^II^O) is a p-type semiconductor [20] that forms monoclinic crystals resulting in specific physical properties, i.e., spin dynamics, electron correlation effects, and high temperature superconductivity. These features make them suitable candidates for application in gas sensors [21], field emission emitters [22], solar energy conversion [23], high temperature super conductors, batteries, and catalysis [24,25,26]. Due to the narrow band-gap energy of Cu^II^O (1.2 eV), researchers are in a continuous quest to investigate their potential applications in various fields, such as photocatalysis, photovoltaics, and photoconduction [27]. These unique properties can be enhanced by synthesis of Cu^II^O nanostructures that have revealed outstanding performance. Several nanostructures of Cu^II^O have been reported, namely, nanoparticle [20], nano-rod [28], nano-wire [22,29], nano-needle [30], and nano-flower [31]. The literature has proposed several methods for synthesis of Cu^II^O NPs in various sizes and shapes such as co-precipitation [32], thermal oxidation [29], combustion [33], and sono-chemical processes [34]. Co-precipitation is a facile method that attracts significant interest in industrial applications due to its low energy consumption, low temperature, and cost-effective approach for bulk production.

Hence, we synthesized peculiar shaped Cu^II^O by using different precursor materials in a simple co-precipitation method. In addition, we also synthesized Fe^II^O, and Fe^III^_2_O_3_ NPs with satisfactory efficiency in a heterogeneous photo-Fenton system. Finally, their photocatalytic results were compared with those of iron(II)-doped copper ferrites (Cu^II^_0.4_Fe^II^_0.6_Fe^III^_2_O_4_) and Cu^II^O/Fe^II^O/Fe^III^_2_O_3_ composite. Two cationic dyes were used as model compounds, namely, methylene blue (MB) and rhodamine B (RhB), for investigating the photocatalytic efficiency of NPs.

## 2. Materials and Methods

### 2.1. Materials

All the chemicals applied were of analytical grade and used without further purification. Copper(II) sulfate (anhydrous), ferric chloride hexahydrate, ammonium iron(II) sulfate hexahydrate, methylthioninium chloride (methylene blue), N-[9-(2-carboxyphenyl)-6-(diethylamino)-3H-xanthen-3-ylidene]-N-ethylethanaminium (rhodamine B), sodium hydroxide, hydrochloric acid, and Fenton reagent (hydrogen peroxide 30% w/w) were obtained from Sigma Aldrich (Budapest, Hungary) and used as received. For pH adjustment, sodium hydroxide or hydrochloric acid were added to the reaction mixture. Ethanol (absolute) and double-distilled water were applied for the purification of the prepared catalysts.

### 2.2. Preparation of Nanoparticles

Metal oxide NPs catalysts were fabricated by using simple (co-)precipitation method as suggested in the literature [35,36]. In this method, solution I was prepared by adding CuSO_4_ metal salt in stoichiometric amount to 20 mL distilled water, while solution II was 5 M NaOH (20 mL) serving as precipitating agent (Table 1). Both solutions (I and II) were sonicated for 30 min at room temperature in an equipment of 40 W power and 115 V voltage (Clean&Bright Inc., Osage Beach, MO, USA). The theoretical stoichiometric compositions of the simple metal oxides and a doped ferrite catalyst are presented in Table 1.

**Figure 1:**
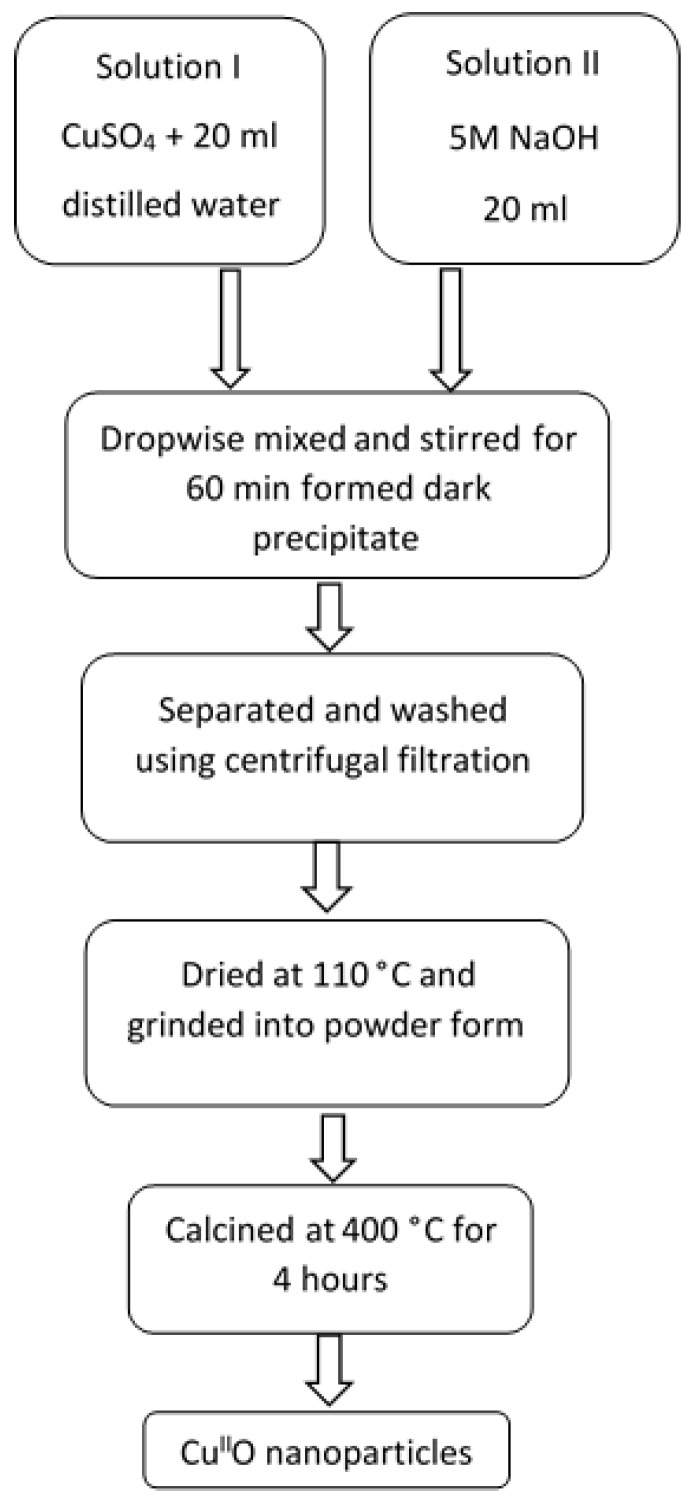
Flow chart representing the steps of Cu^II^O NPs synthesis.

Solutions I and II were mixed together dropwise under continuous stirring, using a magnetic stirrer for 60 min at room temperature (Figure 1). After successful formation of dark precipitates, they were centrifugally separated and purified by using absolute ethanol (twice) and double distilled water (twice). In the centrifugal separation technique, the precipitates were repeatedly re-dispersed and centrifuged twice (at 5500 rpm for 10 min) with ethanol and distilled water to achieve product free from impurities. The obtained purified hydroxide precipitates were dried in oven at 110 °C for 60 min, powdered by using pestle and mortar, and finally calcined at 400 °C for 4 h to obtain the desired powdered catalysts.

From the very low values of the solubility product constants of the corresponding hydroxides (see Text S1 in the Supplementary Materials [37]), we proposed that the total amounts of the metal ions weighed in during the preparation of catalysts were precipitated in the NaOH excess. Moreover, the Cu/Fe ratios in the Cu^II^_0.4_Fe^II^_0.6_Fe^III^_2_O_4_ (after calcination) determined by ICP (inductively coupled plasma) measurements showed 3.9% deviation (Table S1) of the experimental values from the theoretical ones. Similarly, in the case of Fe^II^O, the deviation from the theoretical composition was only 4%, due to the slight oxidation of Fe(II) to Fe(III) during the calcination.

The same synthesis process was applied for the production of Fe^II^O and Fe^III^_2_O_3_ NPs. The preparation process for Cu^II^_0.4_Fe^II^_0.6_Fe^III^_2_O_4_ (designated as NP-3) was already reported in our previous study [36]. The main particle size of the catalyst prepared in this way was in the range of 70–200 nm. In addition, we also made a composite of separated metal oxides (Cu^II^O/Fe^II^O/Fe^III^_2_O_3_) in similar proportion as in Cu^II^_0.4_Fe^II^_0.6_Fe^III^_2_O_4_. The proportion of each metal oxide in composite is presented in Table 2.

The structural elucidation of the powdered catalysts was performed before their application in visible light-induced photocatalytic degradation of MB and RhB.

### 2.3. Characterization of Nanoparticles

SEM images of catalysts were obtained by using a Thermo Scientific scanning electron microscope (SEM model APREO S, Thermo Fisher Scientific Inc., Waltham, MA, USA) equipped with a low-vacuum secondary electron detector. The instrument worked with 20 kV accumulated voltage and 0.80–1.60 mA beam current. For composition and elemental analysis of the catalysts, energy dispersive X-ray (EDX) spectra were obtained by using an EDAX AMETEK instrument (Thermo Fisher Scientific Inc., Waltham, MA, USA) equipped with an octane detector using TEAM software (v.: 4.5, EDAX AMETEK Inc., Mahwah, NJ, USA). The catalysts investigated were uncoated during EDX analysis.

A Philips PW 3710 type powder diffractometer (Philips Analytical B.V., Almelo, The Netherlands) equipped with a graphite diffracted-beam monochromator using CuKα radiations (λ = 0.1541 nm) and a power of 50 kV and 40 mA were used to obtain the X-ray diffraction (XRD) patterns of the catalysts. The XRD patterns were obtained under continuous scan mode with 0.02°/s scanning rate over a 2θ ranging from 10° to 70°. X’Pert High Score Plus software and X’Pert Data Collector (v.: 2.2e (2.2.5), PANanalytical B.V., Almelo, Netherlands) were applied for data evaluations and collections, respectively. Debye-Scherrer equation (Equation (1)) was used to calculate the average crystallite size of catalysts:

(1) $$D=\;\frac{0.9\;\lambda }{\beta cos\theta }$$

where *D* represents the crystallite size; *λ* is the wavelength (0.1541 nm) of the X-ray source; *β* is the line broadening at half the maximum intensity (FWHM) of the average of 3 most intense peaks (in radians); and *θ* is the XRD peak position, i.e., the Bragg angle.

The band-gap energy of catalysts was determined by diffuse reflectance spectroscopy (DRS), using a Perkin Elmer LS50 B spectrofluorometer (PerkinElmer Inc., Waltham, MA, USA), which recorded the scattering spectra of the solid-state samples in the wavelength-range of 250–600 nm. The reflectance (R, Equation (2)) was measured by using barium sulphate as reference material (I_0_). Then, by applying the Kubelka–Munk function (Equation (3)) [38], the values obtained were presented depending on the excitation energy (in eV = electron volt). The band-gap energies (eV) of the catalysts were obtained from the cross-section point of the extrapolated linear portion of the curve on the *x*-axis.

(2) $$R=\frac{I}{I_{0}}$$

(3) $$f\left(R\right)=\frac{{\left(1-R\right)}^{2}}{2R}$$

### 2.4. Measurements of Photocatalytic Activity of the Catalysts

Visible light-induced Fenton degradation of RhB and MB were performed in a quartz cuvette (1 cm pathlength) acting as reactor. The reactor was fitted directly inside a Specord S 600 diode-array spectrophotometer (Analytik Jena GmbH, Jena, Germany). Irradiations were carried out at room temperature by using an Optonica SP1275 LED lamp (GU10, 7 W, 400 Lm, 6000 K, Optonica LED, Sofia, Bulgaria) [36]. The LED lamp-cuvette distance was 4 cm. In this study, several experimental conditions, such as concentration of RhB (1.75 × 10^−5^ mol/L), concentration of MB (1.5 × 10^−5^ mol/L), concentration of NPs (400 mg/L), concentration of H_2_O_2_ (1.76 × 10^−1^ mol/L), reactor temperature (25 ± 2 °C), volume of the reaction mixture (3 mL), and irradiation time (140 min), were kept constant. These parameters were chosen as optimal ones on the basis of our previous study [36].

Control experiments were performed to check the potential self-degradation of dyes under visible light irradiation and in the dark. Some researchers have already confirmed that MB and RhB are stable in the dark but are photosensitive to visible light [39,40,41]. First of all, the reaction rate of the potential photo-induced self-degradation of both dyes were measured. In the next step, the effect of hydrogen peroxide as an oxidant in photo-reaction was studied. Subsequently, the effect of the presence of NPs as heterogeneous photocatalyst was checked. For achieving the adsorption/desorption equilibrium, the mixture of catalyst and dye was kept in a dark room under continuous stirring for 30 min. In the next step, the quartz cuvette was filled with this mixture (3 mL) and set in the sample holder of the spectrophotometer. The photocatalytic reaction was started by putting hydrogen peroxide into the mixture inside the cuvette and opening the window of the light source to irradiate the cuvette containing dye and catalyst NPs. During the irradiation (140 min), the mixture inside the cuvette was continuously homogenized by using a magnetic stirrer (at 240 rpm). The absorption spectra were recorded after suitable time intervals.

The spectral changes observed for both MB and RhB (see later in Section 3.2, Section 3.3 and Section 3.4) confirmed that the photodegradation intermediates as well as the end-products of both dyes showed no appreciable absorption bands in the wavelength range monitored. Thus, in the photocatalysis, the degradation rate could be determined from the decrease of absorbances at maximum wavelengths, i.e., 665 nm for MB and 554 nm for RhB, by application of the Beer–Lambert law, as reported earlier [36], and also described in the SM (Text S2). Baseline correction was applied for elimination of the scattering effects by the solid catalyst particles.

### 2.5. Assessment of Antibacterial Property of NPs

A Thermo ScientificLuminoskan Ascent luminometer (Thermo Fisher Scientific Inc., Waltham, MA, USA) was applied for the measurement of antibacterial property of simple metal oxides and doped NPs. The method was based on the bioluminescence inhibition of the marine, Gram-negative bacterium *Vibrio fischeri* (NRRL-B-11177) purchased from Hach Lange GmbH (Düsseldorf, Germany). The test suspension was prepared according to the manufacturer’s instructions. The lifespan of the test specimen was 4 h after reconstitution. The test protocol followed that reported in the literature [42].

During the evaluation, the results obtained from 2 parallel measurements were averaged and then the relative inhibition_t_ (%) was calculated:

(4) $$Relative\;inhibition_{t}\left(\%\right)=\frac{I_{control\left(t\right)}-I_{sample\left(t\right)}}{I_{control\left(t\right)}}\times 100$$

where *I_control_*_(*t*)_ is the emission intensity of the control samples and *I_sample_*_(*t*)_ = the emission intensity of the test samples.

## 3. Results

### 3.1. Characterization of Synthesized Catalysts

**Figure 2:**
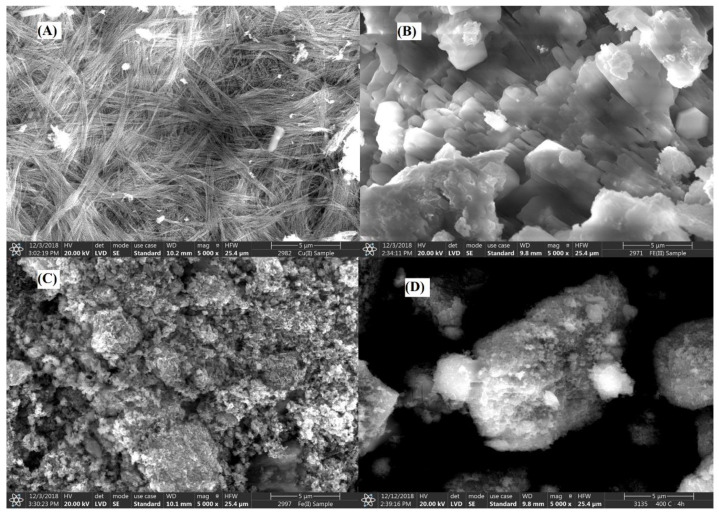
SEM images of synthesized metal oxide and doped ferrite nanoparticles. (**A**) Cu^II^O, (**B**) Fe^III^_2_O_3_, (**C**) Fe^II^O, and (**D**) NP-3.

The detailed description of XRD and Raman spectra of the metal oxides and the ferrite (NP-3) have already been published [36]. Here, the SEM images of the synthesized oxide NPs and NP-3 are shown in Figure 2A–D. Figure 2A revealed about Cu^II^O bead-like uniform structure connected together in threads. Fe^III^_2_O_3_ exhibited rod-like structure with some hexagonal crystals, as shown in Figure 2B, while Fe^II^O possessed a pallet-like structure (Figure 2C). NP-3 had a needle-like structure, embedded into clusters (Figure 2D). SEM images of higher magnification provided a clearer view of the pallet-like structure of Fe^II^O (Figure S1) and needle-like structure of NP-3 (Figure S2). A high degree of agglomeration was observed in Fe^III^_2_O_3_ and Fe^II^O oxides.

**Figure 3:**
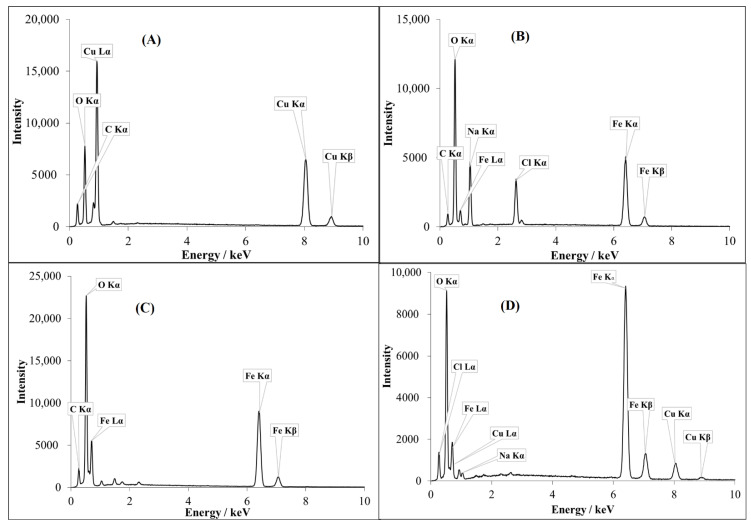
Energy dispersive X-ray (EDX) spectra of simple metal oxides and doped ferrite recorded in scan mode. (**A**) Cu^II^O (**B**) Fe^III^_2_O_3_, (**C**) Fe^II^O, and (**D**) NP-3.

The average intensity values for the constituents present in the NPs were recorded by the EDX spectral analysis in scan mode, while the suspected impurities were identified via spot mode. Cu^II^O contained characteristic peaks of Cu (K_α_, K_β_, and L_α_), O K_α_, and C K_α_, and no significant impurities (Figure 3A), whereas Fe^III^_2_O_3_ contained Fe (K_α_, K_β_, and L_α_), O K_α_, C K_α_, Na K_α_, and Cl K_α_ (Figure 3B). Intensive peaks of Na K_α_ and Cl K_α_ were observed in the EDX spectrum of Fe^III^_2_O_3_, which may have originated from the NaCl cubic crystals (confirmed from SEM). In the case of Fe^II^O, the main peaks were Fe (K_α_, K_β_, and L_α_), C K_α_, O K_α_, and slightly low intensity peaks of Na K_α_ and Cl K_α_ (Figure 3C). The EDX spectrum of doped copper ferrite NP-3 revealed the main peaks of Fe (K_α_, K_β_, and L_α_), O K_α_, Cu (K_α_, K_β_, and L_α_), Na K_α_, and Cl K_α_ (Figure 3D).

Na K_α_ and Cl K_α_ were the dominant impurities in the Fe^III^_2_O_3_, Fe^II^O, and NP-3 catalysts, originating from NaOH and FeCl_3_ during the preparation process. However, aluminum, manganese, silicon, and some traces of sulfur in the form of anion (SO_4_^2−^) were observed in the EDX analysis.

**Figure 4:**
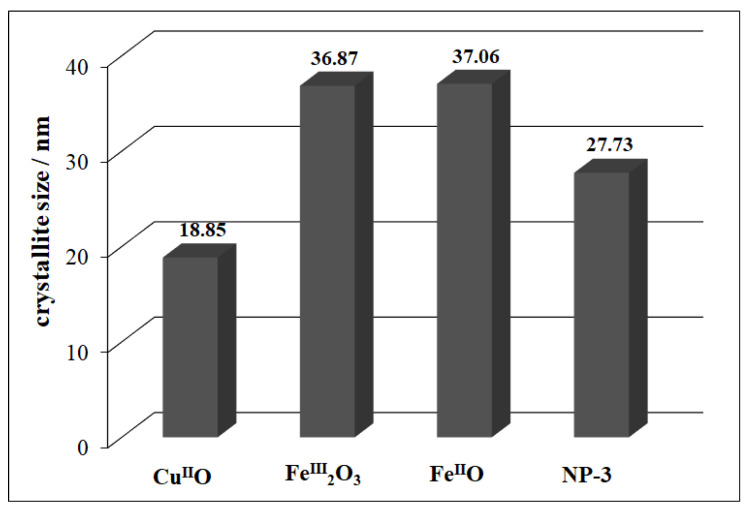
Comparison of the average crystallite sizes of simple metal oxides and NP-3.

The XRD patterns of oxide NPs along with a series of iron(II)-doped copper ferrites were briefly discussed in our earlier work [36]. NP-3 (having the average crystallite size of 27.73 nm) exhibits an inverse spinel structure, where half of the Fe^3+^ ions are in tetrahedral position, while metal ions with +2 oxidation state (Fe^2+^ or Cu^2+^) are in octahedral position [43]. The average crystallite size of the Cu^II^O nanoparticle was found to be 18.85 nm (Figure 4). Comparing the peaks using JCPDS software, we found that the XRD pattern (Figure S3) was well matched with the copper oxide (Cu^II^O) official file “Pdf # 892531”. However, in the case of Fe^III^_2_O_3_, the crystallite size was 36.84 nm and the main characteristic XRD peaks (Figure S3) were in line with the characteristic XRD pattern of hematite with some traces of magnetite. The average crystallite size of Fe^II^O was 37.06 nm. With reference to the XRD patterns [36], we observed wüstite as a major part along with some traces of maghemite in our Fe^II^O sample. This phenomenon occurred as a result of the possible partial oxidation of Fe^2+^ ions to Fe^3+^ during the calcination process.

**Figure 5:**
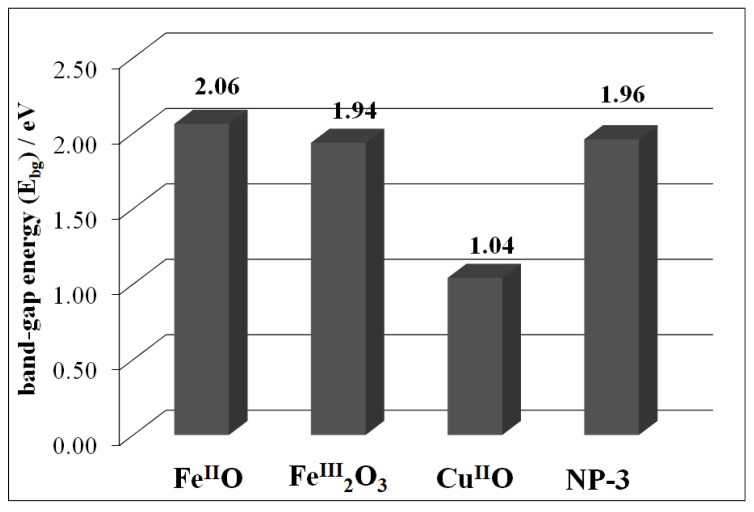
Comparison of band-gap energies (E_bg_) of the simple metal oxides and NP-3.

The detailed DRS spectra were recorded for all metal oxide and ferrite NPs. The band-gap energy (E_bg_) for all of the synthesized NPs were investigated by applying the Kubelka–Munk function (Figure S4). Cu^II^O rendered the lowest band-gap energy (1.04 eV) followed by Fe^III^_2_O_3_ (1.94 eV) and finally Fe^II^O (2.06 eV) (Figure 5).

NP-3 showed the band-gap energy of 1.96 eV. In the earlier published literature, Fe^III^_2_O_3_ displayed a slightly higher band-gap energy (2.0 eV) [44] and the same trend was observed for Cu^II^O (1.2 eV) [45]. Lower band-gap energy is the key to absorb larger portion of sunlight, which is beneficial in photo-Fenton degradation.

### 3.2. Photocatalytic Assessment of Synthesized Catalysts by Using MB

In the first step, control experiments were performed to check the possible self-degradation of MB in the presence or absence of H_2_O_2_ (Fenton reaction), in the dark or light (photo-Fenton reaction) [35]. The combined effect of light and NP-3 on MB degradation was also measured (MB + NPs + light) [36]. Then, the effect of the NPs as heterogeneous photocatalysts was checked (MB + NPs + H_2_O_2_ + light). The results revealed that NPs considerably enhanced the relative rate of MB degradation as compared to the photo-Fenton system (MB + H_2_O_2_ + light) [36].

**Figure 6:**
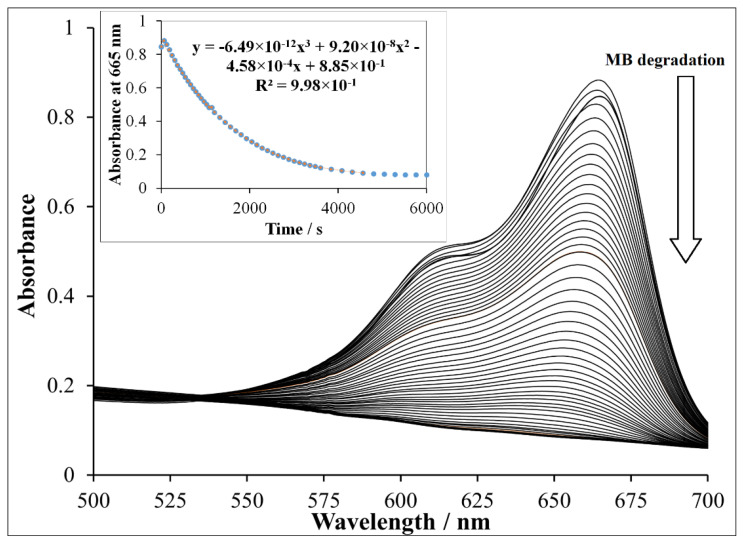
Methylene blue degradation using doped ferrite NP-3. Experimental conditions: concentration of NP-3 = 400 mg/L, concentration of methylene blue (MB) = 1.5 × 10^−5^ mol/L, concentration of H_2_O_2_ = 1.76 × 10^−1^ mol/L, and initial pH = 7.5.

**Figure 7:**
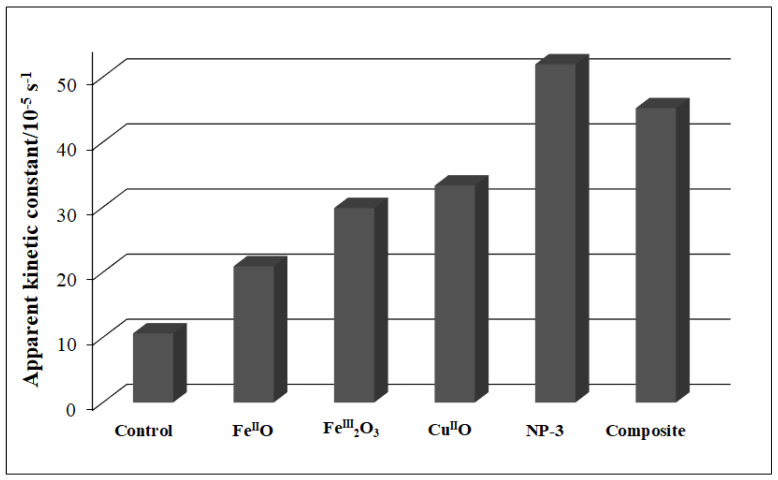
Comparison of apparent kinetic constants of Fe^II^O, Fe^III^_2_O_3_, Cu^II^O, NP-3 (Cu^II^_0.4_Fe^II^_0.6_Fe^III^_2_O_4_), and Cu^II^O/Fe^II^O/Fe^III^_2_O_3_ composite. Experimental conditions: concentration of NPs = 400 mg/L, concentration of MB = 1.5 × 10^−5^ mol/L, concentration of H_2_O_2_ = 1.76 × 10^−1^ mol/L, and initial pH = 7.5.

The detailed spectra changes obtained during the irradiation of the heterogeneous photo-Fenton system containing NP-3 is shown in Figure 6. A pseudo-first-order kinetics was observed from the decay of absorbance at 665 nm (inset of Figure 6). The logarithmic version of this plot (Figure S5) clearly confirmed this observation. It was revealed that almost 100% of MB dye was degraded within about half of the applied irradiation time (140 min) when using NP-3 catalyst. The same type of experiment was carried out with Cu^II^O, Fe^III^_2_O_3_, Fe^II^O, and the Cu^II^O/Fe^II^O/Fe^III^_2_O_3_ composite. The apparent kinetic constants obtained in these experiments were compared (Figure 7). NP-3 exhibited higher degradation efficiency than the Cu^II^O/Fe^II^O/Fe^III^_2_O_3_ composite (the mixture of the simple oxides in the same ratio). The best efficiency of NP-3 regarding the degradation MB (Figure 7) may be attributed to the special needle-like crystalline structure. Among the simple oxides, the highest reaction rate in the case of Cu^II^O may originate from the smaller crystallite size, lower band-gap energy, and highly crystalline structure. Both Fe^III^_2_O_3_ and Fe^II^O NPs rendered lower degradation efficiencies as a consequence of the high degree of agglomeration and comparatively larger crystallite sizes.

### 3.3. Photocatalytic Assessment of Synthesized Catalysts Using RhB

To investigate the photocatalytic activity of catalysts using RhB as model pollutant, we designed the same control experiments (Table 3). The presence of NP-3 enhanced considerably the relative rate of RhB degradation by 346% (Table 3) as compared to (RhB + H_2_O_2_ + light).

**Figure 8:**
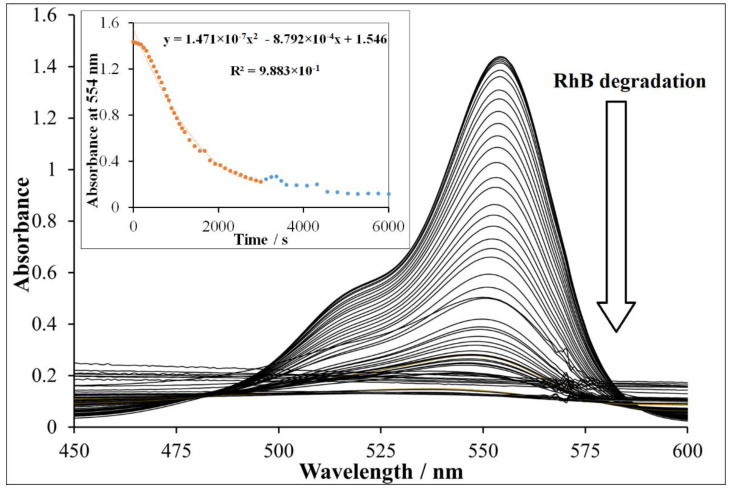
Rhodamine B degradation using Cu^II^O. Experimental conditions: concentration of RhB = 1.75 × 10^−5^ mol/L, concentration of Cu^II^O = 400 mg/L, concentration of H_2_O_2_ = 1.76 × 10^−1^ mol/L, and initial pH = 7.5.

**Figure 9:**
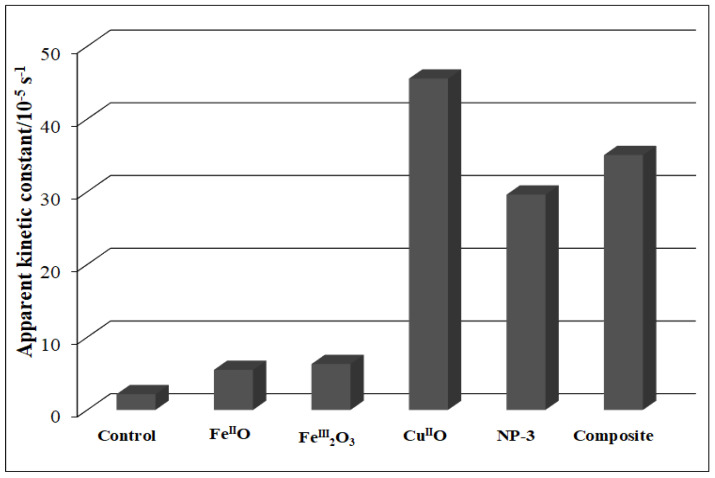
Comparison of apparent kinetic constants of Fe^II^O, Fe^III^_2_O_3_, Cu^II^O, NP-3 (Cu^II^_0.4_Fe^II^_0.6_Fe^III^_2_O_4_), and Cu^II^O/Fe^II^O/Fe^III^_2_O_3_ composite in the photodegradation of RhB. Experimental conditions: concentration of RhB = 1.75 × 10^−5^ mol/L, concentration of NPs = 400 mg/L, concentration of H_2_O_2_ = 1.76 × 10^−1^ mol/L, and initial pH = 7.5.

Figure 8 revealed the detailed spectral changes obtained during the experiment using Cu^II^O as a catalyst. It was observed that almost 100% of RhB was degraded using Cu^II^O in half of the allocated experiment time (inset Figure 8). Moreover, Cu^II^O revealed the highest photocatalytic activity followed by Cu^II^O/Fe^II^O/Fe^III^_2_O_3_ composite and then NP-3 (Figure 9). Lower degradation efficiencies of Fe^III^_2_O_3_ and Fe^II^O NPs were observed as the consequence of larger grain size and higher degree of agglomeration. These results suggest that in this case, the low band-gap energy of Cu^II^O was also an important factor regarding the efficiency. The competitive efficiencies of the combined oxides may be attributed to the synergistic effects of the components (for the composite) and to the advantageous morphology (for NP-3).

### 3.4. UV-Visible Spectral Analysis

**Figure 10:**
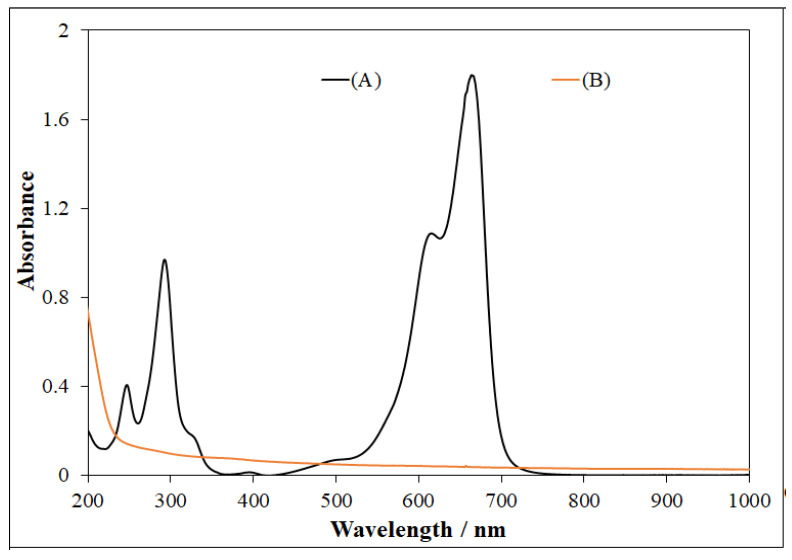
(**A**) Methylene blue spectrum, and (**B**) UV-visible spectrum obtained after MB degradation using NP-3 (Cu^II^_0.4_Fe^II^_0.6_Fe^III^_2_O_4_). Experimental conditions: concentration of MB = 1.5 × 10^−5^ mol/L, concentration of NPs = 400 mg/L, concentration of H_2_O_2_ = 1.76 × 10^−1^ mol/L, initial pH = 7.5, and irradiation time = 140 min.

**Figure 11:**
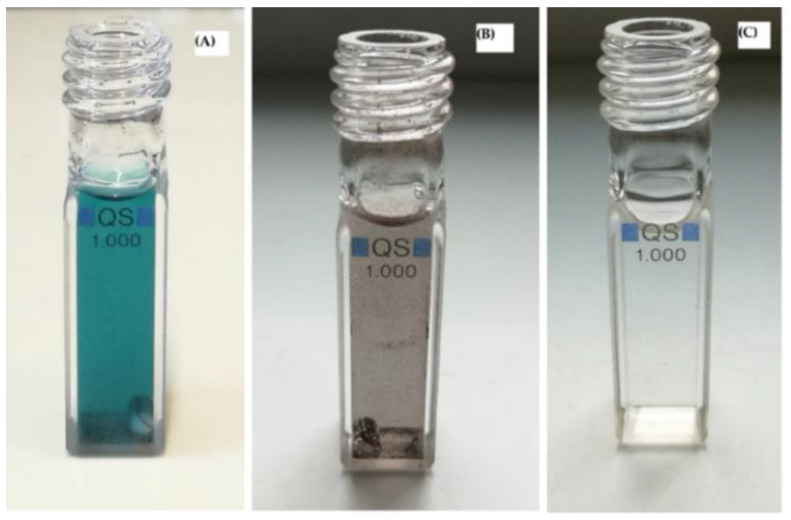
Visual representation of MB degradation in photo-Fenton process in the photo-reactor (cuvette). (**A**) Mixture of MB + NP-3 before photocatalysis, (**B**) MB + NP-3 after photocatalysis, and (**C**) clear solution obtained after separation (centrifugation) of solid catalyst from (**B**).

MB displayed three characteristic peaks at 665, 612, and 292 nm, as shown in UV-visible spectrum (Figure 10). In some cases, MB can be reduced to leuco-methylene blue (λ_max_ = 256, 322 nm) and MBH_2_^+^ (λ_max_ = 232 nm), which are colorless and stable in aqueous medium. Thus, in this photocatalytic system using NP-3 (doped copper ferrite) as catalyst, MB was totally degraded, showing no peaks in the ultraviolet and visible range (Figure 10 and Figure 11).

**Figure 12:**
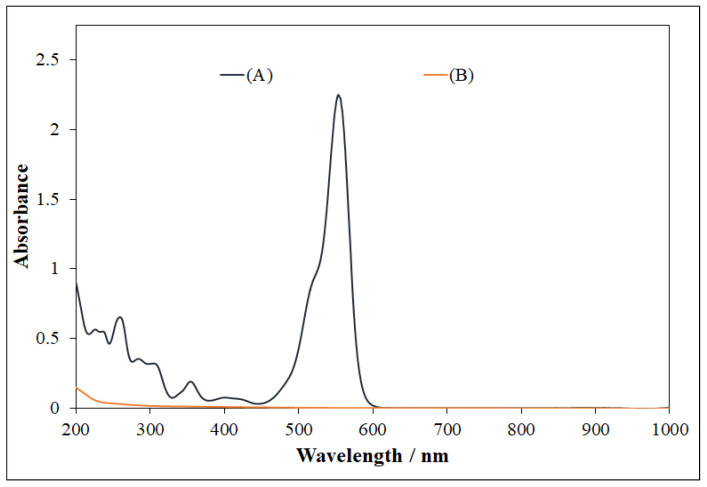
(**A**) Rhodamine B spectrum, and (**B**) UV-visible spectrum obtained after MB degradation using NP-3 (Cu^II^_0.4_Fe^II^_0.6_Fe^III^_2_O_4_). Experimental conditions: concentration of NPs = 400 mg/L, concentration of H_2_O_2_ = 1.76 × 10^−1^ mol/L, concentration of RhB = 1.75 × 10^−5^ mol/L, initial pH = 7.5, and irradiation time = 140 min.

**Figure 13:**
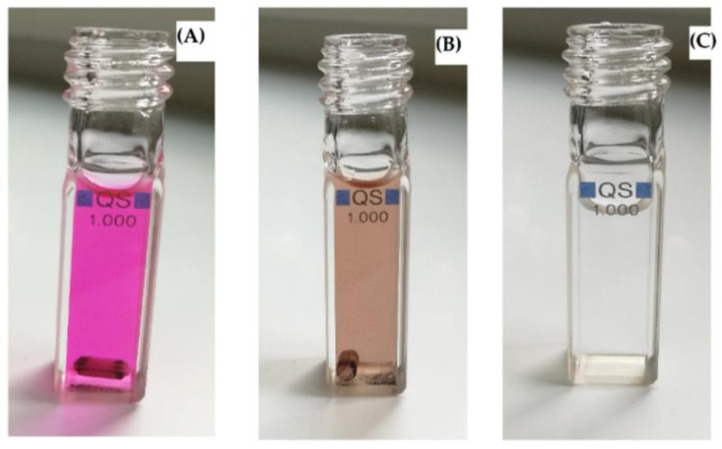
Visual representation of RhB degradation in photo-Fenton process in the photo-reactor (cuvette). (**A**) Mixture of RhB + NP-3 before photocatalysis, (**B**) RhB + NP-3 after photocatalysis, and (**C**) clear solution obtained after separation of solid catalyst from (**B**).

**Figure 14:**
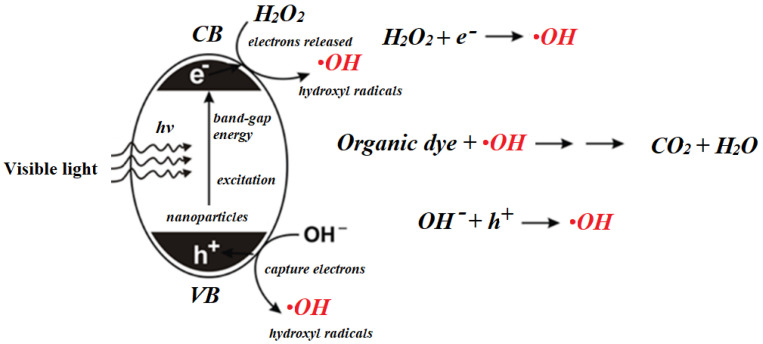
Generalized mechanism for the degradation of organic pollutants using NP-3 (Cu^II^_0.4_Fe^II^_0.6_Fe^III^_2_O_4_).

In the case of RhB, the major peaks were observed at 554, 358, and 262 nm (Figure 12). After photocatalysis at optimized conditions, no peaks were present in the UV and visible regions, which confirmed the complete mineralization of RhB into final products (Figure 12 and Figure 13). A very simple schematic mechanism is proposed for pollutants degradation in Figure 14, which can be explained with reference to the fact that the reactive species produced during the photocatalysis, namely, •OH, h^+^, and •O_2_^–^, oxidize MB [46] and RhB [47] molecules to intermediates with lower molecular weight. In general, in both cases, the first step is N-dealkylation, accompanied or followed by the cleavage of the dye chromophore structure due to the attack of the active species at the central carbon atom. Subsequently, the active species attack the intermediates formed in the previous step, producing smaller open-ring compounds. Finally, the latter compounds are mineralized to water and carbon dioxide.

These results indicate that the degradation efficiencies of Cu^II^O, NP-3, and the composite NPs are competitive with those of TiO_2_-based catalysts [19,28], especially taking into consideration that the latter ones operate under UV irradiation while the heterogeneous photo-Fenton catalysts utilize visible light.

### 3.5. Antibacterial Effect

**Figure 15:**
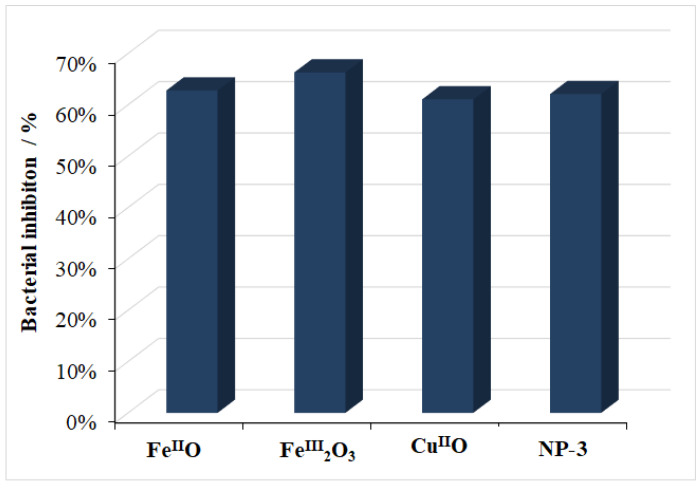
Comparison of bacterial inhibition percentage of Fe^II^O, Fe^III^_2_O_3_, Cu^II^O, and doped NP-3 (Cu^II^_0.4_Fe^II^_0.6_Fe^III^_2_O_4_).

The bacterial inhibitions (%) of simple metal oxides and NP-3 are illustrated in Figure 15. The results revealed that all simple metal oxides and NP-3 showed sufficient antibacterial activities against the Gram-negative bacterium *Vibrio fischeri*. The relative bacterial inhibition was at around 60% in all cases, which suggests a potential application of these oxides in disinfection also. Metal ions from the surface of NPs are absorbed into the cell wall of bacteria, causing damage to its cell membrane by biochemical ways. The presence of solid NPs may also result in biophysical effects due to the immobilization of bacteria, inhibiting their replication processes [48].

### 3.6. Reusability

**Figure 16:**
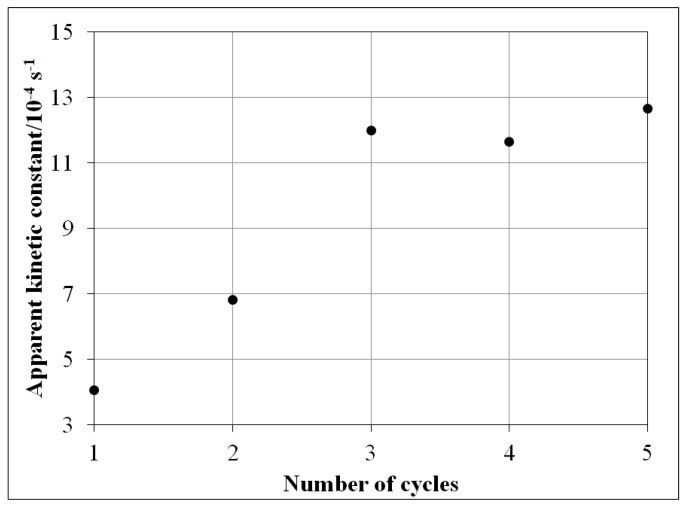
The effect of the reuse of Cu^II^O/Fe^II^O/Fe^III^_2_O_3_ composite catalyst on the apparent kinetic constant of MB. Experimental conditions: concentration of composite = 400 mg/L, concentration of MB = 1.5 × 10^−5^ mol/L, concentration of H_2_O_2_ = 1.76 × 10^−1^ mol/L, pH = 7.5, and irradiation time = 140 min (each cycle).

The reusability results of NP-3 were reported in our previous study [36]. Here, almost the same trend was observed in the case of the Cu^II^O/Fe^II^O/Fe^III^_2_O_3_ composite (Figure 16); the relative degradation efficiency increased until the third cycle. The reaction rate did not change considerably further in the fourth and fifth cycles. In the conventional heterogeneous photo-Fenton system (with iron oxides), the reaction rate would slightly decrease after each cycle. This increase may be attributed to the presence of copper ion in our catalysts, also modifying its surface structure. Such a structural modification may promote the increase of the accessibility of the active sites on the catalyst surface during the first three cycles of application. Moreover, surface morphology may also be changed due to erosion of the superfluous residues from the synthesis.

## 4. Conclusions

This work demonstrated the successful fabrication of novel Cu^II^O, Fe^III^_2_O_3_, and Fe^II^O nanoparticles as well as their composite, and moreover their potential photocatalytic applications in heterogeneous photo-Fenton degradation of rhodamine B and methylene blue. It was confirmed that all metal oxide NPs were active photocatalysts; meanwhile, Cu^II^O revealed excellent degradation efficiency for rhodamine B, while NP-3 revealed the same for methylene blue. Fe^II^O and Fe^III^_2_O_3_ showed lower degradation efficiencies in the case of both model organic dyes due to potential agglomeration and comparatively larger grain size as well as the high band-gap energies. The Cu^II^O/Fe^II^O/Fe^III^_2_O_3_ composite proved to be effective enough for degradation of both organic dyes, probably due to the synergistic effect of the simple components. The better degradation ability of Cu^II^O and NP-3 and Cu^II^O/Fe^II^O/Fe^III^_2_O_3_ composite in the photo-Fenton system could be attributed to a heterogeneous catalysis occurring at the NPs surface, their smaller grain sizes, and special crystalline structure. The latter factor may be the determiner for NP-3, while the low band-gap is the determiner for Cu^II^O. The UV-visible spectra obtained after photocatalysis confirmed the complete mineralization of organic dyes. On the basis of the results of this work, we found that both Cu^II^O and NP-3 as well as the Cu^II^O/Fe^II^O/Fe^III^_2_O_3_ composite could serve as favorable catalysts for the complete mineralization/elimination of toxic organic dyes from textile wastewaters. Interestingly, all metal oxides and NP-3 showed sufficient antibacterial activity against the Gram-negative bacterium *Vibrio fischeri.*

## Figures and Tables

**Table 1:** Metal salts used for the synthesis of oxide nanoparticles (NPs).

Type of NPs	Solution I			Solution II
	CuSO_4_ (g)	Fe(NH_4_)_2_(SO_4_)_2_·6H_2_O (g)	FeCl_3_·6H_2_O (g)	
Cu^II^O	0.798	0	0	5M NaOH
Fe^II^O	0	1.961	0	
Fe^III^_2_O_3_	0	0	2.703	
Cu^II^_0.4_Fe^II^_0.6_Fe^III^_2_O_4_	0.319	1.176	2.703	

**Table 2:** Proportion of metal oxides in the Cu^II^O/Fe^III^_2_O_3_/Fe^II^O composite.

Metal Oxide	Weight (mg/L)
Cu^II^O	54
Fe^III^_2_O_3_	272.5
Fe^II^O	73.5
Total	400

**Table 3:** Design of control experiments for rhodamine B (RhB) degradation. Experimental conditions: concentration of NP-3 = 400 mg/L, concentration of RhB = 1.75 × 10^−5^ mol/L, concentration of H_2_O_2_ = 1.76 × 10^−1^ mol/L, and pH = 7.5.

Description of Experiment	Reaction Rate (M/s)	Relative Rate of Degradation (%)
RhB + light	9.77 × 10^−11^	41
RhB + H_2_O_2_	1.70 × 10^−10^	69
RhB + NPs + light	1.90 × 10^−10^	55
RhB + H_2_O_2_ + light	3.26 × 10^−10^	100.0 (the basis of comparison)
RhB + NPs + H_2_O_2_ + light	1.55 × 10^−9^	346

## Data Availability

The data presented in this study are available on request from the corresponding author. The data are not publicly available due to privacy.

## Supplementary Materials

The following are available online at https://www.mdpi.com/2079-4991/11/1/225/s1. Text S1: Precipitation of metal hydroxides; Table S1: Theoretical and experimental* Cu/Fe ratios of the catalyst (NP-3): Text S2: Determination of the concentrations of the dyes used in this study; Figure S1: SEM image of Fe^II^O showing pallet-like structures; Figure S2: SEM image of NP-3 showing needle-like structures; Figure S3: X-ray diffraction (XRD) diffractograms of iron(II) doped copper ferrite (NP-3) compared to those of the simple oxides of the given metal ions. The characteristic Miller indices indicated for the compounds the standards of which were earlier studied by XRD are taken from the International Centre for Diffraction Data; Figure S4: Kulbelka-Munk function for determination the band-gap energy (E_bg_) of NP-3 and simple metal oxides; Figure S5: The logarithm of the absorbance at 665 nm vs. time plot for the degradation of MB (see the inset of Figure 6). Experimental conditions: conc. of NP-3 = 400 mg/L, conc. of MB = 1.5 × 10^−5^ mol/L, conc. of H_2_O_2_ = 1.76 × 10^−1^ mol/L, and initial pH = 7.5.
