# Supplementary material for: Comparing the Degradation Potential of Copper(II), Iron(II), Iron(III) Oxides, and Their Composite Nanoparticles in a Heterogeneous Photo-Fenton System
Source: Nanomaterials (Basel). 2021 Jan 16;11(1):225. doi: 10.3390/nano11010225 (PMC7829782; doi:10.3390/nano11010225)
Supplement: Supplementary file 1 [file nanomaterials-11-00225-s001.pdf]

Supplementary Materials

for

Comparing the degradation potential of copper(II), iron(II), iron(III) oxides, and their composite nanoparticles in heterogeneous photo-Fenton system

Asfandyar Khan[1,2], Zsolt Valicsek[1], Ottó Horváth[1,*]

[1] Department of General and Inorganic Chemistry, Faculty of Engineering, University of Pannonia, Veszprém – Hungary
[2] Department of Textile Processing, National Textile University, Faisalabad – Pakistan
* Correspondence: horvath.otto@mk.uni-pannon.hu e-mail@e-mail.com; Tel.: +36-88-624000 / 6049

Content **Page Nr.**

Text S1, Table S1, Text S2 2

Figures S1-2 3

Figures S3-4 4

Figure S5 5

Text S1. Precipitation of metal hydroxides

The values given in the manuscript for the metal oxides and $Cu^{II}_{0.4}Fe^{II}_{0.6}Fe^{III}_2O_4$ catalyst samples are not only theoretical values. Since 5M NaOH was applied in excess for the precipitation, the total amounts of the metal ions weighed in were precipitated, due to the very low values of the solubility product constants (K_{sp}) [41]:

$Fe(OH)_2$ $8.00x10^{-16}$ M^3
$Fe(OH)_3$ $2.79x10^{-39}$ M^4
$Cu(OH)_2$ $2.20x10^{-20}$ M^3

On the basis of these K_{sp} values, the theoretical concentrations in the solution phase were

$Fe(OH)_2$ $3.20x10^{-17}$ M
$Fe(OH)_3$ $2.23x10^{-41}$ M
$Cu(OH)_2$ $8.80x10^{-22}$ M

Besides, no formation of hydroxo complexes occur in these systems.

Table S1. Theoretical and experimental* Cu/Fe ratios of the catalyst (NP-3).

**$Cu^{II}_{0.4}Fe^{II}_{0.6}Fe^{III}_2O_4$ NP-3**	
Theoretical Cu/Fe ratio	0.154
Experimental Cu/Fe ratio*	0.148
Deviation (%)	3.90

*Determined by ICP measurements.

Text S2. Determination of the concentrations of the dyes used in this study

The actual concetrations of the dyes were determineed by using the Beer Lambert law (Eq. (1)),

$$A_{\lambda,t} = \varepsilon_\lambda c_t \ell \qquad (1)$$

where A is the absorbance as the function of wavelength (λ, in unit "nm"), and time (t, in unit "s"), ε is the molar absorbance of dye ($M^{-1}cm^{-1}$) as the function of wavelength, c is the concentration of dye (M) in the solution and ℓ is the path length of the cuvette (cm).

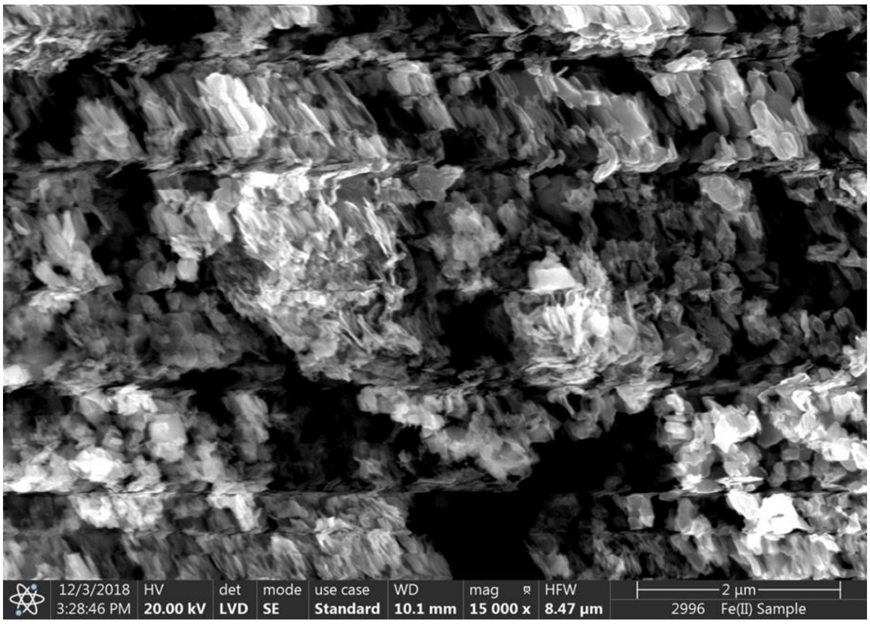

Figure S1. SEM image of $Fe^{II}O$ showing pallet-like structures

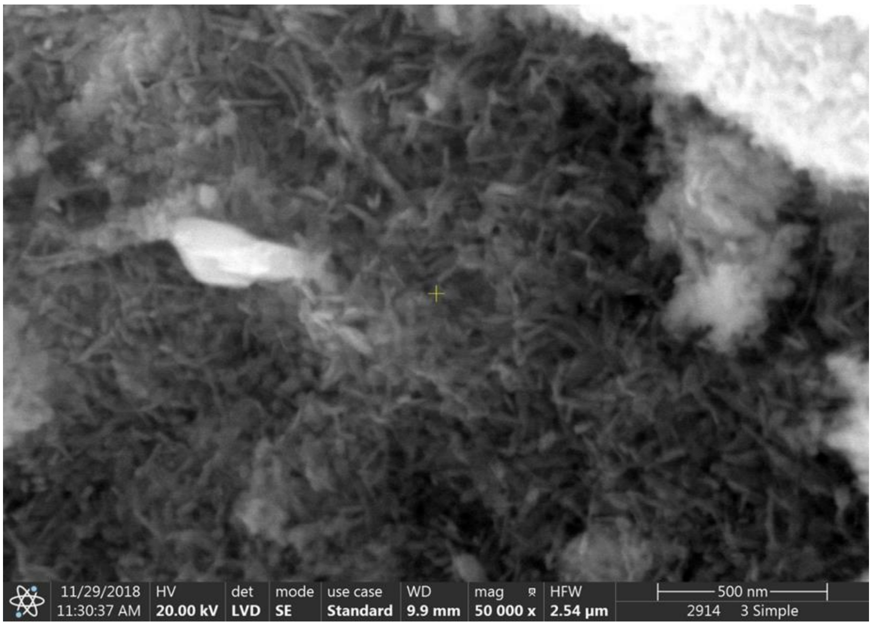

Figure S2. SEM image of NP-3 showing needle-like structures

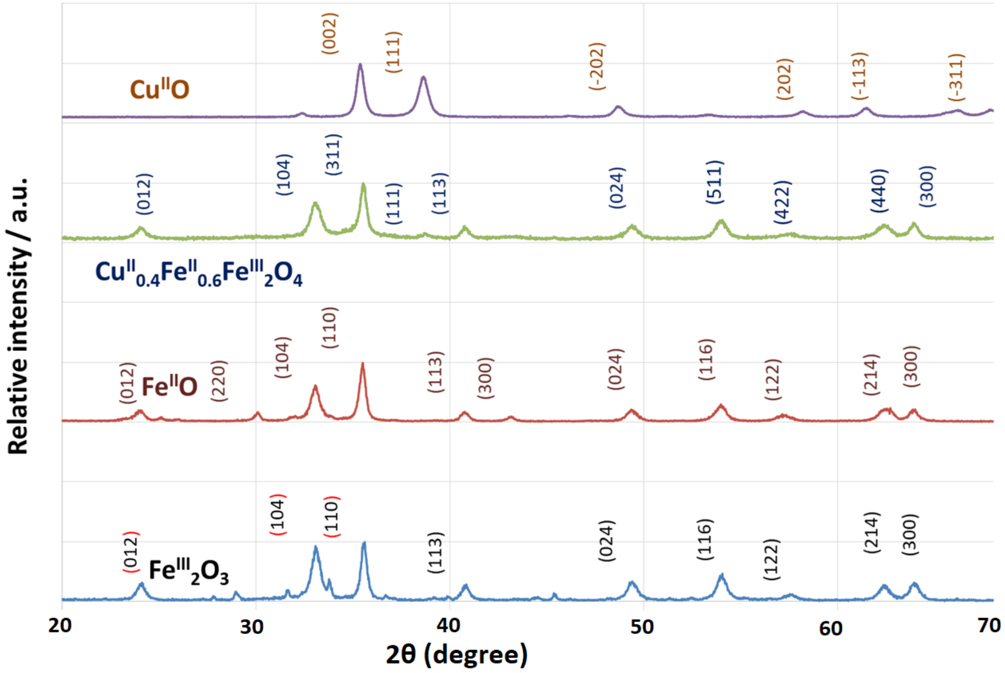

Figure S3. . X-ray diffraction (XRD) diffractograms of iron(II) doped copper ferrite (NP-3) compared to those of the simple oxides of the given metal ions. The characteristic Miller indices indicated for the compounds the standards of which were earlier studied by XRD are taken from the International Centre for Diffraction Data.

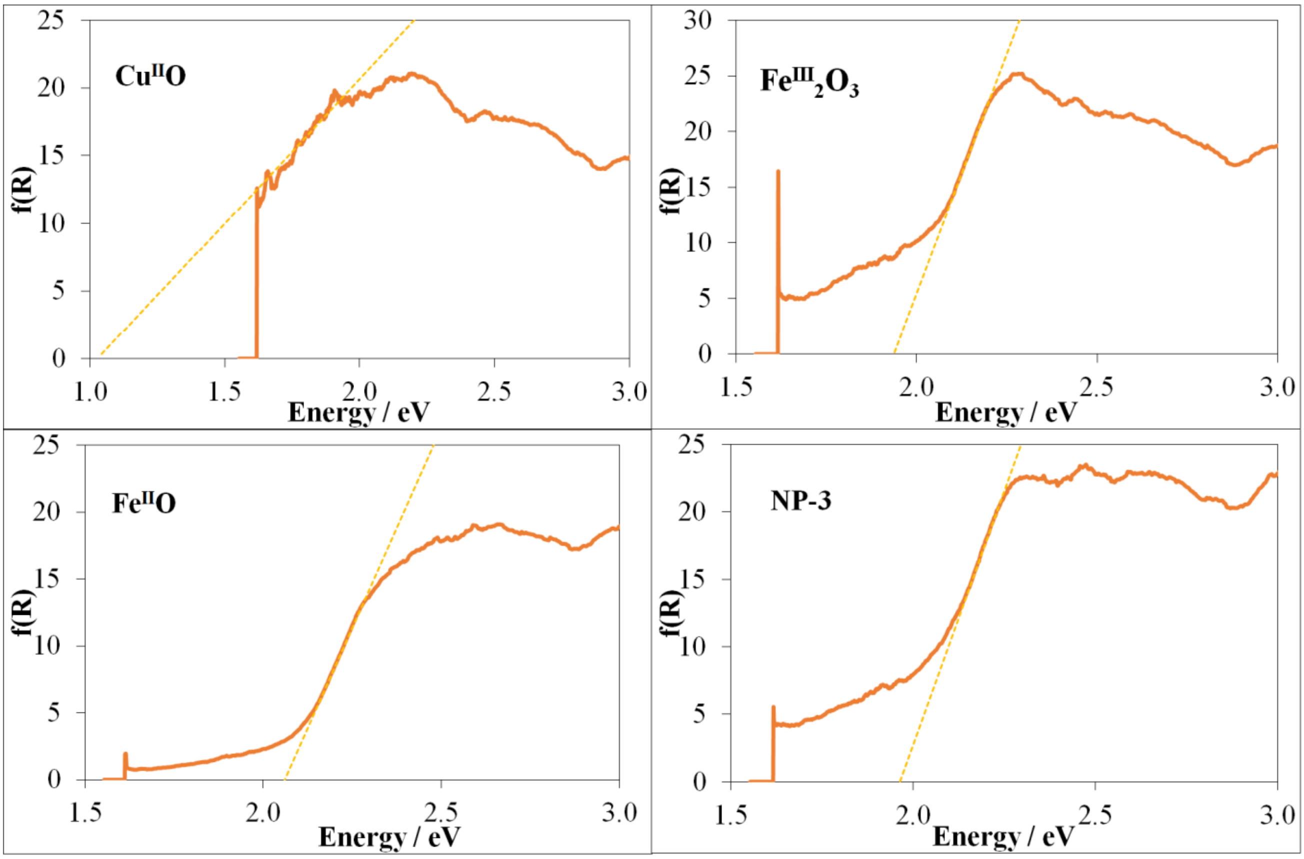

Figure S4. Kulbelka-Munk function for determination the band-gap energy (E_{bg}) of NP-3 and simple metal oxides.

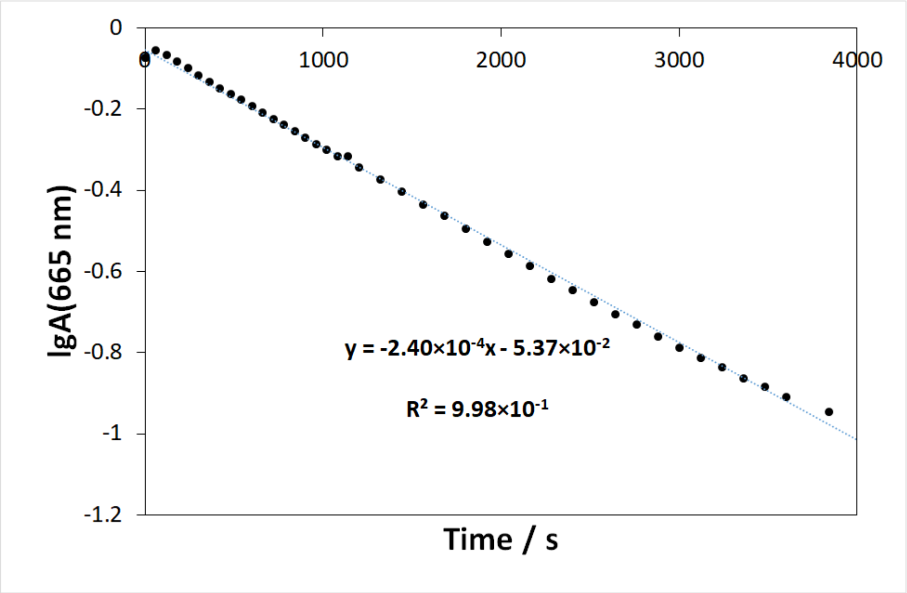

Figure S5. The logarithm of the absorbance at 665 nm vs. time plot for the degradation of MB (see the inset of Fig. 6). Experimental conditions: conc. of NP-3 = 400 mg/L, conc. of MB = 1.5×10^{-5} mol/L, conc. of H_2O_2 = 1.76×10^{-1} mol/L, and initial pH = 7.5.
